# Transcriptomic profile of the most successful *Mycobacterium tuberculosis* strain in Aragon, the MtZ strain, during exponential and stationary growth phases

**DOI:** 10.1128/spectrum.04685-22

**Published:** 2023-10-26

**Authors:** Jessica Comín, Elena Campos, Jesús Gonzalo-Asensio, Sofía Samper

**Affiliations:** 1 Instituto Aragonés de Ciencias de la Salud, Zaragoza, Spain; 2 Universidad de Zaragoza, Zaragoza, Spain; 3 Fundación IIS Aragón, Zaragoza, Spain; 4 CIBER de Enfermedades Respiratorias, Madrid, Spain; Iowa State University, Ames, Iowa, USA

**Keywords:** transcriptome, mycobacteria, iron metabolism

## Abstract

The *Mycobacterium tuberculosis Zaragoza* (MtZ) strain has produced the largest outbreak in Aragon Community, Spain, as it started in the 90s and continues today, contributing with more than 240 cases of tuberculosis, around 10% of the TB cases. Previous studies were carried out to identify the reasons for its successful spread capacity among the population. Some single nucleotide polymorphisms (SNPs) in genes considered as virulence factors were found, as well as an oversecretion of PE_PGRS factors. Different copies of IS*6110* have been localized, and a transcriptomic study was carried out to study how the different IS*6110* copies could affect the adjacent gene expression. In this work, we analyzed the transcriptomic profile of the MtZ, strain trying to delve into the mechanisms behind its success. We found that ESX-1 system is upregulated during both exponential and stationary growth phases *in vitro*, as well as the virulence pathways of cholesterol degradation and peptidoglycan biosynthesis. In addition, MtZ has the iron uptake genes downregulated in exponential growth phase (mycobactin biosynthesis and IrtAB transporters), while the iron storage could be upregulated. The *desA3* gene transcription was interrupted by the insertion of an IS*6110* copy. This gene codes for a stearoyl-coenzyme A (CoA) Δ9 desaturase involved in the production of oleic acid, which is necessary for the metabolism of the bacteria. In spite of this, although MtZ strain has an initial long lag phase growing in liquid media, it is capable of achieving normal growth, suggesting the existence of an alternative route for obtaining oleic acid.

**IMPORTANCE:**

Aragon Community suffered, during the first years of the beginning of this century, a large outbreak caused by the MtZ strain, producing more than 240 cases to date. MtZ strain and the outbreak have been previously studied from an epidemiological and molecular point of view. In this work, we analyzed the transcriptomic profile of the strain for better understanding of its success among our population. We have discovered that MtZ has some upregulated virulence pathways, such as the ESX-1 system, the cholesterol degradation pathway or the peptidoglycan biosynthesis. Interestingly, MtZ has downregulated the uptake of iron. Another special feature of MtZ strain is the interruption of *desA3* gene, essential for producing oleic acid. Although the strain takes a long time to grow in the initial culture media, eventually it is able to reach normal optical densities, suggestive of the presence of another route for obtaining oleic acid in *Mycobacterium tuberculosis*.

## INTRODUCTION

*Mycobacterium tuberculosis* is the causative agent of tuberculosis (TB) disease. In 2019, TB was the 13th cause of death worldwide, and the World Health Organization estimated that 10 million people developed the disease in 2020 and around 1.5 million died because of it (1). Aragon Region, Spain, has an incidence of around 10 cases per 100,000 inhabitants, and since 2004, the surveillance protocol carried out includes the genotyping of all *M. tuberculosis* isolates.

The largest outbreak in Aragon was caused by a strain called *Mycobacterium tuberculosis Zaragoza* (MtZ), described in previous works (2 – 4). The outbreak started in the 90s and continues today, with more than 240 cases reported to date and representing around 10% of the total cases in our community. MtZ belongs to the modern lineage L4.10, with an undefined spoligotype (SIT-773) and 12 IS*6110*. Previous analyses of this strain detected eight SNPs in genes considered as virulence factors, as well as an increase in the secretion of PE_PGRS family proteins (4). Absence of secretion of these factors has been related with an increase in virulence in the Beijing family (5), but how oversecretion, as observed in MtZ, affects virulence remains unknown. Furthermore, we carried out the transcriptomic study using three MtZ isolates that differed in the location of an IS*6110* copy in order to identify differences in the expression of the adjacent genes next to IS*6110*, where no significant differences were detected (4).

The aim of this work is to provide the whole transcriptome analysis of the MtZ strain in order to shed light on the virulence and pathogenic mechanisms that allowed MtZ to become such a successful strain in Aragon.

## RESULTS

**Fig 1:**
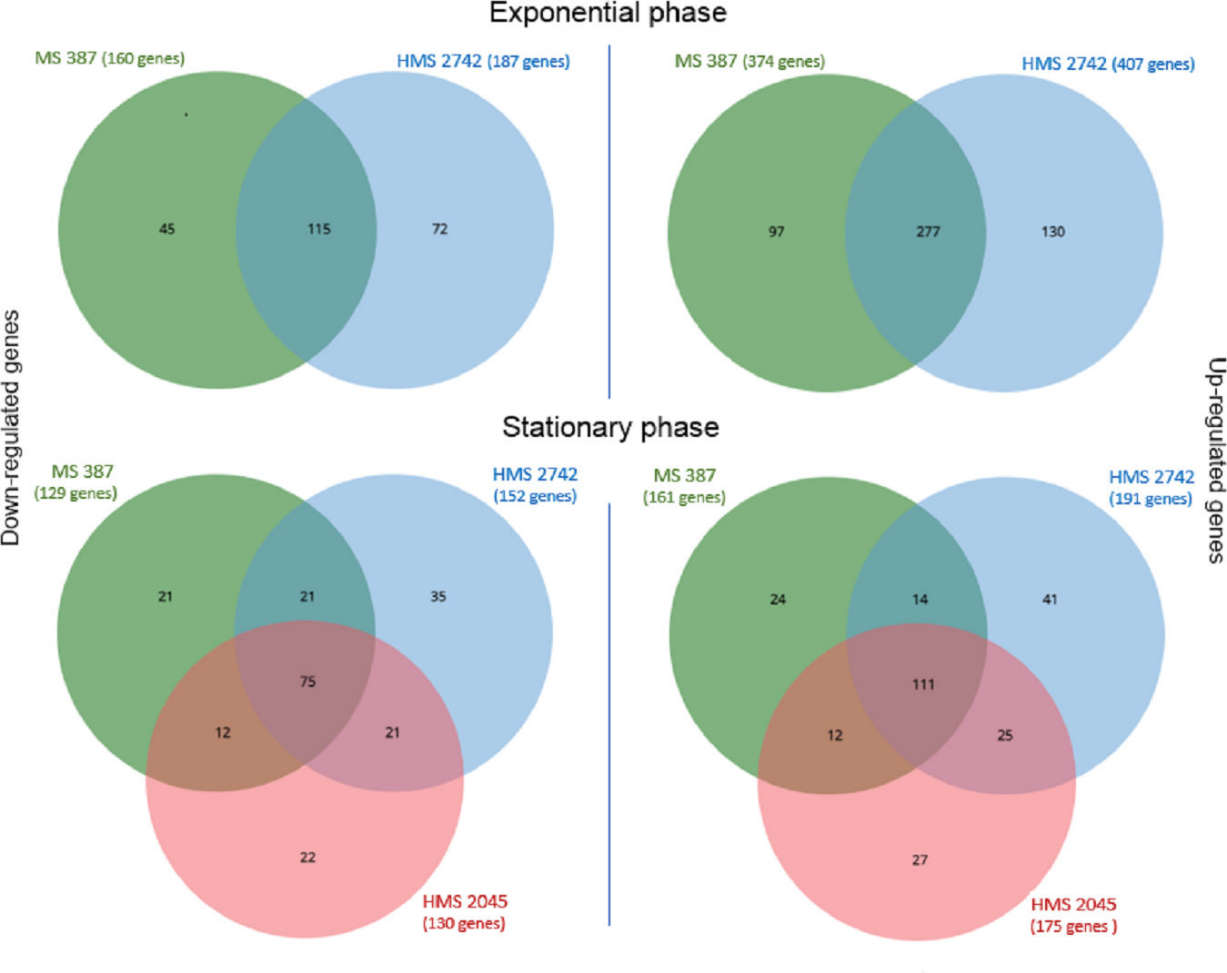
Venn diagrams showing the number of differentially expressed genes for the strains analyzed. The common genes for each condition are listed in Table S2.

The differential expression analysis results for each strain in exponential and stationary phases compared to the H37Rv controls are shown in the supplemental table (Table S1). The three MtZ strains isolates analyzed are MS 387 (isolated in 1995, one of the first isolates detected), HMS 2742 (isolated in 2012 with a 10-kb deletion affecting part of the CRISPR region), and HMS 2045 (isolated in 2007 with an extra IS*6110* in *dnaA:dnaN*, only analyzed in the stationary growth phase). The differentially expressed genes were split into upregulated and downregulated for each of the three MtZ isolates analyzed (Table 1). The common differentially expressed genes were extracted using Venn diagrams (Fig. 1). Only the common differentially expressed genes found among the three MtZ strains were taken into account (Table S2). The common differentially expressed genes were analyzed with Cytoscape software using BiNGO plugin tool to group them into functional categories. A summary of the results can be found in Tables 2 and 3 of the present work. More information can be found in the supplemental materials (Table S3).

**TABLE 1:** Differentially expressed genes for the MtZ isolates analyzed vs H37Rv strain

Differentially expressed genes for the three MtZ isolates analyzed				
	Strains			
	MS 387	HMS 2742	HMS 2045	Common genes
Exponential phase				
Downregulated	160	187		115
Upregulated	374	407		277
Stationary phase				
Downregulated	129	152	130	75
Upregulated	161	191	175	111

**TABLE 2:** Upregulated and downregulated pathways and processes during exponential growth phase of the MtZ strain *^a^*

Exponential growth phase		
Upregulated pathways and processes	Downregulated pathways and processes	
Cholesterol degradation	Import of iron-bound mycobactin	
Phosphate assimilation	Mycobactin and siderophore biosynthesis	
Survival within host macrophages	Response to iron starvation	
Host immune system modulation	Pathogenic lipid synthesis	
Transcriptional regulators	Propionate degradation	
Removal of signal peptides	Lipid metabolism	
Biosynthesis of some fatty acids	Toxin-antitoxin systems	
Peptidoglycan biosynthesis	Sulfur metabolism	
Host cell invasion	Biosynthesis of some amino acids	
Toxin-antitoxin systems	Transcriptional regulators	
Protection of oxidative and metal damage	Translation	
Transporters	Mycolic acid composition and permeability of the envelope	
Iron storage		
Integrity of the cell wall		
DNA repair		
Protein folding		
Persistence in the host		
Growth regulation		
Viral latency		
Lipid catabolism and degradation		
Degradation of some particular amino acids		
Molybdopterin biosynthesis		
Purine and pyrimidine biosynthesis		

^*a*^ More information can be found in Supplementary materials (Table S3).

**TABLE 3:** Upregulated and downregulated pathways and processes during stationary growth phase of the MtZ strain*^a^*

Stationary growth phase	
Upregulated pathways and processes	Downregulated pathways and processes
ESX-1 secretion system	Mycolic acid composition and permeability of the envelope
ESX-2 secretion system	Transcriptional factors
Biosynthesis and secretion of siderophore	Phospholipid metabolism
Lipid metabolism	Lipid metabolism
Molybdopterin biosynthesis	Toxin-antitoxin systems
Transporter	
Transcriptional regulators	

^*a*^ More information can be found in the supplemental materials (Table S3).

### Upregulated genes in the MtZ strain during exponential growth phase *in vitro*

**Fig 2:**
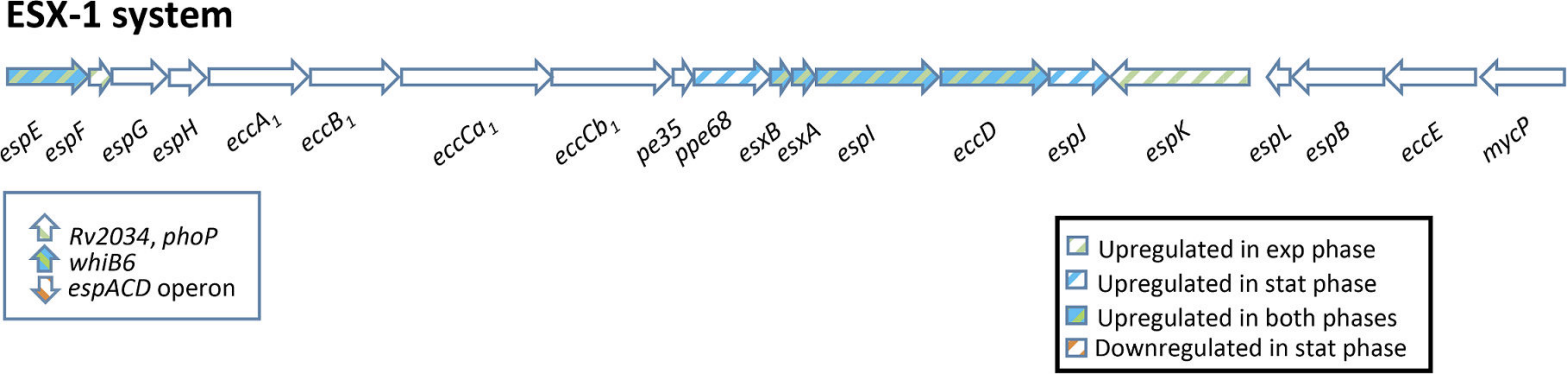
Schematic view of the ESX-1 genomic region showing altered transcription in the MtZ strain compared with H37Rv. The system seems upregulated in both in exponential and stationary growth phases. Some genes related with ESX-1 system, but not belonging to the operon, were included in an independent square: *esp*ACD is a pathogenic island in H37Rv; *Rv2034*, *phoP*, and *whiB6* regulate the expression of ESX-1 system. exp, exponential; stat, stationary,

BiNGO tool (Table S3.1) showed that the MtZ strain had upregulated genes involved in pathogenesis routes as cholesterol degradation (*kshA*, *cyp125*, *choD, hsaE*, *hsaF*, and *hsaG* genes), phosphate assimilation (*regX3* and *phoP*), survival of mycobacteria within host macrophages (*pknG*, *PE_PGRS33*, *esxA*, *esxB*, and *pirG*), modulating host immune system (*PE_PGRS11*, *PE_PGRS30*, *pknK*, *esxA*, and *esxB*), transcriptional regulators (*sigD*, *rpfC*, and *Rv0386*), removal of signal peptides (*lspA*), biosynthesis of some fatty acids (*pks5*, required for full virulence during host infection; *pks6*; and *pks7*) and peptidoglycan biosynthesis (*Rv1433*, *ldtA*, *murG*, and *ald*). Besides, many genes of the ESX-1 secretion system (*espE*, *espF*, *esxA*, *esxB*, *espI*, and *espK*) (Fig. 2) and of the ESX-2 system (*eccD2*, *mycP2*, *eccE2*, and *eccA2*) were upregulated. In addition, some *mce* operon genes, involved in host cell invasion, were upregulated (*mce3A*, *mce3C*, *mce3D*, and *lprK*) as well as some toxin-antitoxin systems (*vapB22*, *vapB17-vapC17*, *vapB36-vapC36*, and *vapB41*).

The category “response to stimulus” had many upregulated genes involved in different processes including protection of oxidative and metal damage (*ahpC*, *PE_PGRS11*, *ephB*, *mymT*, *Rv3378*, and *bpoA*), transporters (*Rv1687c*, *Rv1739c*, *Rv1986*, *ctpG*, secG, *Rv1686c*, *yrbE3A*, *mmpS2*, *mmpL2*, *mmpL10*, *mmpL12*, *glnH*, *narU*, and *pstS3*), transcriptional regulators (*Rv0792c*, *Rv1985c*, *Rv2034*, *Rv2621c*, *Rv2884*, *Rv3249c*, *cmtR*, and *Rv0386*), iron storage (*bfrB*), integrity of the cell wall (*fbpC*, *pimF*, *ripB*, and *ald*), DNA repair (*Rv0921*, *Rv3394c*, *Rv3395c*, and *dnaE2*), protein folding (*groES*), persistence in the host (*Rv2557*), and growth regulation (*higB* and *mazF5* toxins). In addition, many genes related with viral latency were upregulated (*Rv1577c*, *Rv1586c*, *Rv2658c*, *Rv2659c*, and *Rv2651c*).

In addition to genes involved in cholesterol degradation and peptidoglycan biosynthesis, many other genes involved in lipid metabolism were upregulated in MtZ strain: fatty acid biosynthesis (*pks4*, *pks5*, *pks6*, *pks7*, *pks8*, *pks9*, *pks17*, *pks18*, *acpM*, *fadD15*, *accA1*, and *Rv1894c*), lipid catabolism and degradation (*lipL*, *alkB*, *Rv1592c*, *mutB*, *scoA*, *scoB*, *Rv3502c*, and *Rv1075c*), and other lipid metabolic processes (*Rv2277*, *papA3*, *Rv0111*, *Rv3378c*, and *Rv2499c*). The degradation of some particular amino acids, especially the ones with uncharged polar side chains, was also upregulated: *ald* (L-alanine), *accA1* and *accD1* (L-leucine), *scoB* (valine, leucine, and isoleucine), *bkdA* and *bkdB* (branched-chain amino acids), and *Rv1188* (L-proline). Finally, some genes involved in the molybdopterin biosynthesis pathway (*moaC3*, *moaA1*, *Rv3324A*, and *moaX*), as well as genes involved in the purine and pyrimidine biosynthesis (*purT*, *purU*, *pyrB*, and *pyrC*), were likewise upregulated. A summary of the affected pathways and processes can be found in Table 2.

### Downregulated genes in MtZ during exponential growth phase *in vitro*

**Fig 3:**
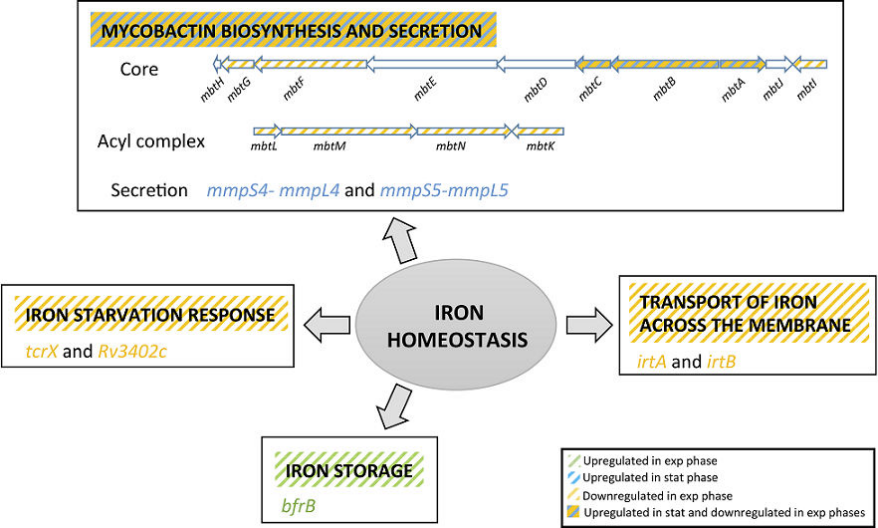
Affected iron homeostasis in MtZ strain. Four pathways were affected during exponential and stationary phases, including mycobactin biosynthesis (both operons *mbt-1*, responsible for the mycobactin core synthesis, and *mbt-2*, for the acyl complex that finishes the synthesis of the mycobactin), iron starvation response (*tcrX* and *Rv3402c* genes), transport of iron across the mycobacterial membrane (*irtA* and *irtB* genes) and iron storage (*bfrB*). exp, exponential; stat, stationary.

Using BiNGO tool (Table S3.2) *irtA* and *irtB* genes were shown to be downregulated in MtZ strain. These genes codify for an ABC transporter complex involved in the import of iron-bound mycobactin. Genes involved in the mycobactin and siderophore biosynthetic pathway (*mbtA*, *mbtB*, *mbtC*, *mbtF*, *mbtG*, *mbtI*, *mbtK, mbtL*, *mbtM*, and *mbtN*) and response to iron starvation (*tcrX* and *Rv3402c*) were also downregulated (Fig. 3). *Rv1085c*, a hemolysin-like protein involved in virulence, was also downregulated.

Another downregulated gene was *whiB3*, a redox-sensitive transcriptional regulator that maintains intracellular redox homeostasis by regulating catabolic metabolism and polyketide biosynthesis. It regulates pathogenic lipid synthesis, coordinating propionate flux and the storage of lipid triacylglycerol. This gene controls the expression of *pks2*, involved in sulfolipid-1 biosynthesis, and *pks3*, both downregulated in MtZ strain. *papA1* gene, also involved in sulfolipid-1 biosynthesis, was downregulated. *prpC*, *prpD*, and *prpR* genes, involved in the propionate degradation route, were downregulated, probably as consequence of *whiB3* downregulation. Many other genes related to lipid biosynthesis (*ppsC*, *ppsD*, *desA3*, *Rv1760*, and *accA2*) and lipid degradation (*Rv1096*, *fadE35*, *fadE12*, and *echA18*) are downregulated. *desA3* gene, involved in the synthesis of oleic acid, is interrupted by an IS*6110*, and *Rv1760*, involved in the synthesis of triacylglycerol, is absent in MtZ strain due to an IS*6110*-mediated recombination. Besides, *Rv3083-lipR-Rv3085*, part of the *mymA* operon and required for maintaining the appropriate mycolic acid composition and permeability of the envelope on its exposure to acidic pH, were absent in MtZ strain as this deletion is characteristic of all L4.8 strains (RD219). *ppe57*, which plays a key role in regulating innate and adaptive immune responses through human toll-like receptor 2 (TLR2), was also absent in the MtZ strain, as well as *wag22*, described to have fibronectin-binding activity that could mediate bacterial attachment to host cells, and it is thought to be expressed during infection.

In addition, MtZ strain has some downregulated toxin-antitoxin systems (*vapC19* and *vapB47-vapC47*), as well as some genes related to sulfur metabolism (*cysH*, *sirA*, and *che1*). The biosynthesis of some amino acids such as methionine (*mmuM*, *metH*, and *metK*) and histidine (*Rv3137* and *hisE*) are downregulated, and many transcriptional regulators (*rpfE*, *oxyS*, *Rv1049*, *Rv2912c*, and *Rv1287*). Finally, genes involved in mRNA translation also appear to be downregulated (*rplN*, *rpsF*, and *rplJ*). A summary of the affected pathways and processes can be found in Table 2.

### Upregulated genes in MtZ strain during stationary growth phase *in vitro*

The analysis performed by BiNGO tool showed that some routes associated with pathogenesis were upregulated in MtZ strain in the stationary phase (Table S3.3). During stationary phase, *whiB6*, *esxA*, and *esxB* genes were upregulated similarly to the exponential growth phase, as well as *espE*, *espI*, *espJ*, and *ppe68* (together with *pe35*, which stimulates the secretion of IL-10 and MCP-1 from human macrophages, via the interaction with human TLR2 and *eccD*, all of them part of the ESX-1 secretion system (Fig. 2). In addition, we found upregulation of the *whiB5* gene, coding for a redox-responsive transcription factor that plays a role in immunomodulation and reactivation after chronic infection and induces the transcription of ESX-2 and ESX-4. *mycP2* gene, belonging to the ESX-2 secretion system, was also found to be upregulated. The sigma factor *sigD*, whose expression decreases during hypoxia, was upregulated. The route of biosynthesis and secretion of siderophores (*mbtA*, *mbtB*, *mbtC*, *mmpS4- mmpL4*, and *mmpS5-mmpL5*), essential for virulence, was upregulated (Fig. 3). Finally, *hbhA* gene (required for extrapulmonary dissemination and responsible of inducing mycobacterial aggregation), *icl1* (involved in the persistence and virulence through glyoxylate cycle), and *rpfA* and *rpfE* (factors that stimulate resuscitation of dormant cells with peptidoglycan hydrolytic activity) were also upregulated.

Many genes involved in lipid biosynthesis (*pks4*, *pks5*, *pks18*, *ino1*, *papA3*, *Rv3740c*, *fadD15*, *Rv0111*, *umaA*, and *mmpL10*), lipid degradation (*fadD3, Rv3551*, and *ipdC*, all related to cholesterol metabolism, and *Rv1592c*), and lipid metabolism (*fadB2*, *fadE5*, *Rv2251*, *Rv2277*, *fadE35*, and *lipU*) were upregulated in MtZ strain. Similar to the exponential growth phase, the molybdopterin biosynthesis pathway (*moaC3*, *moaA1*, *Rv3324A*, and *moaX*) was upregulated, along with some toxin-antitoxin systems (*vapC36*, *vapC1*, and *vapB44*). Many transporters, some of them with an unknown substrate, as *Rv1435c*, *Rv1218c*, *efpA*, *mmpL10*, and *Rv1463*, were also upregulated. Finally, in addition to those already described, other transcriptional regulators (*Rv2612c*, *Rv3093c*, *Rv0474*, *Rv0196*, *ethR*, *Rv1219c*, and *Rv3413c*) were upregulated. A summary of the affected pathways and processes during stationary growth phase can be found in Table 3.

### Downregulated genes in MtZ strain during stationary growth phase

The operon *espA-espC-espD*, considered a pathogenicity island in H37Rv and involved in the ESX-1 secretion system, was downregulated in MtZ in the stationary growth phase (Fig. 2) (Table S3.4). Also, the operon *Rv3083-lipR-Rv3085-Rv3087-tgs4*, required for maintaining the appropriate mycolic acid composition and permeability of the envelope upon exposure to acidic pH, was downregulated. *Rv3083*, *lipR*, and *Rv3085* are absent in MtZ as is RD219, characteristic of L4.8 strains. Many transcriptional factors were downregulated in this growth phase: *rpfD*, *mce1R*, *Rv0195*, *tcrX*, and *Rv0516c*. Regarding lipid metabolism, fatty acid biosynthesis was clearly downregulated (affecting *Rv1760*, absent in MtZ because of an IS*6110*-mediated recombination, together with *Rv3233c*, *accA2*, *Rv3720*, *Rv1184c*, *fcoT*, and *pks3*), as well as phospholipid metabolism (*cdh* and *mmpL8*), lipid degradation (*fadE12*), and others (*Rv0100*, *fadD10*, and *echA7*). Finally, the toxin-antitoxin *vapB47-vapC47* was also downregulated. A summary of the affected pathways and processes can be found in Table 2.

## DISCUSSION

The results obtained in RNAseq regarding the MtZ strain, related to H37Rv used as control, has allowed us to analyze the upregulated and downregulated processes and metabolic pathways of this successful tuberculosis strain. Parts of the ESX-1 system seem upregulated in MtZ strain (Fig. 1), in both exponential and stationary growth phases. This system is essential for virulence in *M. tuberculosis*, required for the rupture of the phagosome and the liberation of bacteria in the cytoplasm, mainly mediated by *esxA-esxB* heterodymer. ESX-1 has been also linked with necrosis/apoptosis induction on the host cell, granuloma formation, and cell-to-cell spread (6 – 8). Several ESX-1 secretion-associated proteins as EspE and EspI (both in exponential and stationary growth phases), EspF and EspK (exponential growth phase), the transmembrane protein EccD1 (stationary phase), PPE68 (stationary phase), and the major ESX-1 effector protein EsxA and its chaperone EsxB (exponential and stationary growth phases) are upregulated. *whiB6* gene, identified as a transcriptional regulator of the ESX-1 system (9), is also upregulated. It has been observed that H37Rv presents a single-nucleotide insertion in the promotor of *whiB6* (9), specifically in the PhoP-binding region, which was related with the downregulation of the ESX-1 system in H37Rv in comparison to clinical strains (10, 11), what could explain why the system appeared upregulated in MtZ as the comparison was carried out using H37Rv as control. However, MtZ strain has gotten upregulated *phoP* gene (about two fold change) and *Rv2034* gene, which positively regulates transcription of *phoP*, both in the exponential growth phase, suggesting that MtZ really could have its virulence increased mediated by the *phoP*-ESX-1 system. However, in the stationary phase, MtZ has downregulated *espA-espC-espD* genes, an operon considered a pathogenicity island in H37Rv (7, 12) and which is normally secreted along *esxA-esxB* genes. Other *esp* genes, such as *espE* and *espF*, are homologous to these genes, so they could be replacing the pathogenicity island roll in the ESX-1 system.

MtZ strain also presented part of the ESX-2 system to be upregulated (*eccD2*, *mycP2*, *eccE2*, and *eccA2* genes), recently described to be involved in phagosome permeabilization and bacterial scape (13). This operon has been shown to be regulated by *whiB5* gene (14). However, *whiB5* was not upregulated in the MtZ strain during exponential growth phase, when we observed operon overexpression. *whiB5* was upregulated in stationary growth phase, when only *mycP2* gene was upregulated in MtZ strain. Besides, we did not observe the overexpression effect in any of the genes supposedly controlled by *whiB5*. These observations make us speculate that upregulation of part of the ESX-2 system has another cause different from *whiB5*.

Iron is essential for *M. tuberculosis* as it plays important roles in vital biologic processes, including electron transport (15). To obtain iron from the environment, *M. tuberculosis* produces mycobactins, essential for the *in vivo* growth and survival of the pathogen (16). The genes that synthesize these mycobactins are organized in two operons, *mbt-1*, including *mbtA-mbtJ* genes, responsible for the synthesis of the core structure of the mycobactin molecule, and *mbt-2*, including *mbtK-mbtN* genes, responsible for incorporating the hydrophobic aliphatic side chain onto the mycobactin backbone (17). Besides, both operons are regulated by *ideR*, so that in the presence of iron, *ideR* acts as a repressor of the mycobactin biosynthesis (18), promoting iron storage through the expression of *bfrA* and *bfrB* (19). What we observed during the exponential growth phase of the MtZ strain is that many genes of *mbt-1* and all genes of *mbt-2* were downregulated, while *bfrB* was upregulated (Fig. 2), mimicking high iron level in the environment, so that mycobactins were not required. In this sense, the bacteria would have enough iron to store and thus avoid its toxicity. This observation would be supported by the downregulation of *irtA* and *irtB*, encoding for IrtAB cytoplasmatic iron transporter (20). Considering that the growth conditions were the same for the control strain H37Rv and MtZ, it suggests that this iron response must be somewhat specific for the MtZ strain. In the stationary growth phase, where low iron levels are expected, MtZ overexpressed some mycobactin genes (*mbtA*, *mbtB*, and *mbtC*) as well as *mmpS4-mmpL4*/*mmpS5-mmpL5*, described as forming a new siderophore export system for mycobactins (21). This iron incorporation defect could be compensated with the heme uptake, not depending on mycobactins (22), although none of the genes involved in this pathway was upregulated in MtZ strain.

During both exponential and stationary growth phases, MtZ strain presented upregulation of the molybdopterin biosynthesis pathway (*moaC3*, *moaA1*, *Rv3324A*, and *moaX*). MtZ has an IS*6110* inserted at point 3668723 (referred to *Mycobacterium bovis* AF2122/97 genome), adjacent to the operon, and it is in forward direction, whereas the genes of this operon are in reverse direction. Therefore, overexpression of the operon does not seem to be the result of IS*6110* acting as a promotor, described in other works (23 – 25). Besides, *moaA1* is outside of the operon. Among the molybdenum enzymes in mycobacteria, we found the nitrate reductase, encoded by the *narGHIJ* locus (26) and considered an important virulence factor because nitrate is described to enhance survival during inhibition of respiration (27), and also the carbon monoxide dehydrogenase, which plays a role in the protection of the bacteria from nitrosative stress during infection (28), and the biotin sulfoxide reductase. Biotin plays an important role in the citric acid cycle, cell signaling, epigenetic regulation, and chromatin structure (29) and is also required for obtaining malonyl-CoA, used for mycolic acid biosynthesis, and the degradation of many pyridine derivatives is catalyzed at the first step by molybdenum enzymes (28). *sigD*, a transcriptional regulator, was upregulated during exponential and stationary growth phases in the MtZ strain. This gene is suggested to play an important role in optimal growth and survival both under starvation and nutrient replete conditions, and many genes have been identified as being regulated directly or indirectly by *sigD* (30). We found some of the *sigD*-regulated genes (*rpfC*, *groES*, *fbpC*, *papA3*, and *fadD15*) to be upregulated in MtZ during the exponential and stationary growth phases *in vitro*.

Lipids from the host are used by *M. tuberculosis* as carbon source, allowing mycobacterial persistence (31). The main lipids used by *M. tuberculosis* are fatty acids and cholesterol, transformed into bacterial products that mediate pathogenesis, replication, drug tolerance, and virulence (32). One of these products is propionyl-CoA, which can be used for central metabolism as part of the methylcitrate cycle (33, 34) or for the synthesis of virulence-associated cell wall lipids as part of the methylmalonyl pathway and polyketide biosynthesis (35). During exponential growth phases, the MtZ strain appeared to be redirecting its metabolism to obtain propionyl-CoA, as we observed upregulation of genes involved in the degradation of the A and B rings of cholesterol at some stages (*kshA*, *hsaF*, and *hsaG* genes), as well as the b-oxidation of the side chain (*cyp125*) and also the degradation of branched chain amino acids (*bkdA* and *bkdB*), another source of propionyl-CoA. Besides, once propionyl is obtained, it is redirected toward the methyl-malonyl pathway (*mutB* gene is upregulated in MtZ) and from there to the synthesis of virulence lipids of the cell wall (many *pks* genes are upregulated, both in exponential and stationary growth phases). In the stationary growth phase, the degradation of the C and D rings of cholesterol was upregulated (*fadD3* and *ipdC* gene) as was the transformation of propionyl-CoA into succinate and pyruvate through the methylcitrate cycle, which was actually downregulated in the exponential growth phase (*prpC* and *prpD* genes).

Peptidoglycan is important for maintaining cell shape, and it fortifies the plasma membrane against the osmotic pressure of the cytoplasm (36). During stationary and exponential growth phases, the peptidoglycan remodeling was upregulated in MtZ through *ldtA*, *murG*, and *Rv1433* genes (all involved in the peptidoglycan synthesis), *ripB* (required for normal separation of daughter cells after cell division and cell wall integrity and for host cell invasion), and *rpfC* (with peptidoglycan hydrolytic activity). In addition, MtZ had upregulated some other genes involved in the maintenance of the cell wall integrity: *fbpC* (antigen 85 protein, catalyzes the transfer of mycolic acids to cell wall arabinogalactan) and *pimF*. The synthesis and translocation of polyacyltrehalose (*pks4*, *papA3*, and *mmpL10*) and phthiocerol dimycocerosates (PDIM) (*pks8*, *pks9*, and *pks17*) seemed to be also upregulated in the exponential growth phase, while the polyacyltrehalose synthesis seemed downregulated during stationary growth phase (*Rv1184c* and *pks3*). However, the synthesis of sulfolipids (*pks2* and *papA1* genes) was downregulated. This altered composition of the cell wall could be due to the downregulation of *whiB3*, demonstrated as being responsible for modulating the biosynthesis of complex virulence lipids (37). In addition, metyl-malonyl concentration has been suggested to regulate the biosynthesis of both sulfolipids and PDIM, so that in the absence of PDIM, biosynthesis of sulfolipids increases (38); conversely, the reverse situation is possible. That is what we observed in the MtZ strain: the downregulation of sulfolipids but the upregulation of PDIM.

One important fact in MtZ strain is the inactivation of *desA3* gene due to an IS*6110* insertion (point 3606308 in H37Rv genome) (3, 4). *desA3* encodes a stearoyl-CoA Δ9 desaturase that produces oleic acid, a precursor for mycobacterial membrane phospholipids and triglycerides (39) and considered essential (40) because it is the only enzyme with this activity described in *M. tuberculosis* (41). In *Mycobacterium smegmatis*, a knockout of the homologous gene of *desA3* resulted in a decrease of oleic acid of about 40%–50% but remained viable on solid media, whereas in liquid media, the knockout strain showed an aberrant growth with a long lag phase until the bacteria started to grow, not reaching the optic density of the wild-type strain in any case (41). The authors of the study concluded that there were other enzymes that complemented the activity of *desA3*. This aberrant growth is exactly what we observe when the MtZ strain is grown in liquid media. The fact that the strain remained viable may suggest that there could be some oleic synthesis by an unknown enzyme, as oleic acid is essential for mycobacterial growth.

The main limitation of this study is that the interpretation could be biased by comparing MtZ strain against a laboratory H37Rv. In addition, transcriptomic alone is not enough to extract definitive conclusions.

In summary, we have analyzed the transcriptomic differences of the MtZ strain compared to H37Rv and found some differences that could explain the success of this tuberculosis strain among our population in Aragon. More studies such as metabolomics, proteomics, or cellular studies would be needed to complement the results obtained and improve our understanding of *M. tuberculosis* behavior.

## MATERIALS AND METHODS

### Strains and growing conditions

Three different MtZ isolates were selected for the transcriptomic study: MS 387 (clinical isolate of 1995), HMS 2045 (isolated in 2007 with an extra IS*6110* located in *dnaA:dnaN*), and HMS 2742 (isolated in 2012 with a deletion of ∼10 kb, including the genes from *Rv2816c* to the point of insertion of IS*6110* in *Rv2823c* because of an IS*6110*-mediated recombination) (42). H37Rv strain was used as control. Three replicates of each strain were used. The strains, stored at −80°C were thawed, and culture was started a in 7H9 medium supplemented with 10% (vol/vol) albumin-dextrose-catalase. A second culture was started under the same conditions until DO = 0.6–0.7 for exponential growth phase and DO = 1.2–1.3 for stationary growth phase. Strain HMS 2045 was eliminated from the exponential phase study because the analysis of the results indicated that it was a different strain than expected, possibly due to inoculum failure.

### RNA extraction and sequencing

The RNA was extracted using the FastPrep homogenizer and the chloroform-isoamilic alcohol-isopropanol method from 10 mL of bacterial culture (43). The samples were sent to the STAB-VIDA company (Caparica, Portugal; https://www.stabvida.com) for the transcriptomic analysis. The library construction of cDNA molecules was carried out using a ribosomal depletion library preparation kit. The generated DNA fragments were sequenced in the lllumina Novaseq platform using 150-bp paired-end sequencing reads. The analysis of the generated raw sequence data were carried out using CLC Genomics Workbench version 12.0.3. The high-quality sequencing reads were mapped against the reference genome *M. tuberculosis* H37Rv (NC_000962.3).

### Bioinformatic analysis

The analysis of the transcriptomes were carried out independently for each RNA isolate, and subsequently, the average of the three replicates was performed to obtain the average values. Those genes that were expressed significantly different in the three strains with respect to H37Rv were used in the study. The common differentially expressed genes were extracted using Venn diagram available online (https://www.biotools.fr/misc/venny). Integrative Genome Browser software was used for a visual study of the transcriptome (44) using the WIG files of the transcriptome. Cytoscape software and BiNGO tool (supported by the U.S. National Institute of General Medical Sciences) were used to group the genes in different metabolic pathways. Mycobrowser (https://mycobrowser.epfl.ch/), Uniprot (https://www.uniprot.org/), and KEGG PATHWAY database (https://www.genome.jp/kegg/pathway.html) were used to find information of the genes and proteins of interest.

## Data Availability

The raw data of the transcriptomic samples presented in this study can be found in the National Center for Biotechnology Information repository. The BioProject accession number is PRJNA1021783, biosamples: SAMN37570867–SAMN37570886.

## SUPPLEMENTAL MATERIAL

Table S1 spectrum.04685-22-s0001.xlsx Differentially expressed genes vs H37Rv of each strain and condition studied reference strain.

Table S2 spectrum.04685-22-s0002.xlsx Common_dif_expressed_genes.

Table S3 spectrum.04685-22-s0003.xlsx Genes classified by functional pathways.

ASM does not own the copyrights to Supplemental Material that may be linked to, or accessed through, an article. The authors have granted ASM a non-exclusive, world-wide license to publish the Supplemental Material files. Please contact the corresponding author directly for reuse.
